# Obesity and motor skills among 4 to 6-year-old children in the united states: nationally-representative surveys

**DOI:** 10.1186/1471-2431-12-28

**Published:** 2012-03-15

**Authors:** Katia Castetbon, Tatiana Andreyeva

**Affiliations:** 1 Rudd Center for Food Policy and Obesity, Yale University, New Haven, CT, USA; 2 Unité de surveillance et d'épidémiologie nutritionnelle, Institut de veille sanitaire, Université Paris 13, Bobigny, France

**Keywords:** Child Development, Childhood Obesity, Gross Motor Skills, Fine Motor Skills, National Survey, BMI references

## Abstract

**Background:**

Few population-based studies have assessed relationships between body weight and motor skills in young children. Our objective was to estimate the association between obesity and motor skills at 4 years and 5-6 years of age in the United States. We used repeated cross-sectional assessments of the national sample from the Early Childhood Longitudinal Survey-Birth Cohort (ECLS-B) of preschool 4-year-old children (2005-2006; n = 5 100) and 5-6-year-old kindergarteners (2006-2007; n = 4 700). Height, weight, and fine and gross motor skills were assessed objectively via direct standardized procedures. We used categorical and continuous measures of body weight status, including obesity (Body Mass Index (BMI) ≥ 95^th ^percentile) and BMI z-scores. Multivariate logistic and linear models estimated the association between obesity and gross and fine motor skills in very young children adjusting for individual, social, and economic characteristics and parental involvement.

**Results:**

The prevalence of obesity was about 15%. The relationship between motor skills and obesity varied across types of skills. For hopping, obese boys and girls had significantly lower scores, 20% lower in obese preschoolers and 10% lower in obese kindergarteners than normal weight counterparts, *p *< 0.01. Obese girls could jump 1.6-1.7 inches shorter than normal weight peers (*p *< 0.01). Other gross motor skills and fine motor skills of young children were not consistently related to BMI z-scores and obesity.

**Conclusions:**

Based on objective assessment of children's motor skills and body weight and a full adjustment for confounding covariates, we find no reduction in overall coordination and fine motor skills in obese young children. Motor skills are adversely associated with childhood obesity only for skills most directly related to body weight.

## Background

Despite recent progress towards stabilization in the prevalence of childhood overweight and obesity in the U.S. [1] and other countries [2-4], many children still have excessive body weight. In 2007-2008 in the U.S., around 17% of 2-to-19-year-old children had a body mass index (BMI) at or above the 95^th ^percentile of the U.S. growth charts while 32% were overweight or obese (BMI ≥ 85^th ^percentile) [1]. Childhood obesity has considerable adverse consequences for children's physical health, persistence of obesity into adulthood and health later in life [5]. In response to these patterns, prevention of childhood obesity has become a national priority in many countries.

Childhood obesity may lead to impaired cognitive and physical development [6], which can translate into deleterious social and economic consequences such as social exclusion, diminished school performance, and ultimately poorer labor market outcomes [7]. Mechanisms of these effects are still incompletely understood. One mechanism involved in these observations could be through the inhibiting effect of obesity on children's physical development. Overweight and obese children unable to successfully engage in physical challenges may resist participating in physical activities and overall learning solicitations. Furthermore, parents, caregivers and teachers may be less likely to encourage obese children to engage in physical activity based on their perceptions that the child has limited physical abilities [8]. Impaired physical development could trigger a cycle of physical activity avoidance and reduced social interactions, which could lead to further reduction in physical fitness of obese children [9]. This, in turn, could contribute to negative health and weight outcomes [10,11].

Prior research on the relationship of childhood obesity with motor skill development has produced mixed results. Two studies showed more limited motor skills (gross and fine skills evaluated together) among obese boys compared to normal weight peers, but these results were not shown in girls [12,13]. Several studies assessing overall gross motor skills found impaired skills in obese children regardless of gender [14-18] or only in boys [19]. This was also the case for object-control skill components in both girls and boys [14,17]. For fine motor skills, results are more mixed due to a lower number of studies, which usually suggest no negative association with obesity until 9 years of age [20]. Comparisons across these cross-sectional studies are limited due to differences in the methods used, especially for motor skill assessment. In addition, previous studies were based on rather small sample sizes (from one hundred [14,15,17,18] to less than 700 children [16], except for one large survey in Germany) [19] and/or biased samples (with no random selection in representative samples).

As a result, available data on the relationship between childhood obesity and motor skill development at early ages remains inconclusive [21]. One study using a longitudinal design and controlling for reverse causality showed that childhood overweight contributed to a delay in motor development, but the survey sample was limited to low-income African-American infants from 3 to 18 months of age [22]. Furthermore, an interventional study of children in an obesity treatment intervention showed that reduced mean body weight was accompanied by improved gross motor coordination performance [23].

Our study tests the hypothesis that fine and gross motor skills are inversely associated with BMI z-scores and obesity in young American children. We estimate cross-sectional associations of fine and gross motor skills with BMI z-scores and obesity accounting for individual differences in the learning and family environment and socio-demographic characteristics of preschoolers (4 year-olds) and kindergarteners (5-6 year-olds) residing in the United States.

## Methods

### Sample

We used repeated cross-sections of a national sample of U.S. children from the Early Childhood Longitudinal Survey-Birth Cohort (ECLS-B), a nationally-representative longitudinal study of U.S. children born in 2001 conducted by the National Center for Education Statistics (NCES) [24,25]. Access to the ECLS-B data is allowed only to researchers who are granted a restricted-use data license. In addition, an approval has been obtained from the Office for Human Research Protections (OHRP) of the Yale University (n°0808004141).

Children were assessed at 9 months of age (about 10 700 children out of 14 000 initially sampled), and at 2, 4 and 5-6 years of age. The survey had a complex design selecting counties or combinations of counties as primary sampling units and stratifying them by region, median household income, proportion of minority population, and metropolitan/non-metropolitan area (38 strata in total). Births were sampled from the National Center for Health Statistics (NCHS) vital statistics system. The survey excluded children born to mothers younger than 15, or those who were adopted or died before 9 months.

### Data collection

The survey collected data from multiple sources, including direct assessment of children at their homes, computer-assisted interviews with parents (usually the mother; the father or another guardian in less than 5% of cases), and surveys of child care providers and teachers. Signed informed consent was obtained from the respondent before the parent interview began.

We used data collected at preschool age or prior to entering kindergarten (August 2005-June 2006) and at kindergarten age (September 2006-March 2007).

#### Anthropometry

The ECLS-B trained interviewers measured children's height and weight using a standardized protocol [26]. With children dressed in light clothing and without shoes, height was measured using a portable stadiometer and weight was measured with a digital scale. Measurements were taken twice and the average for each measurement was used. BMI was calculated as weight (kg) divided by height (m) squared and converted into BMI z-scores and percentiles for age and sex based on the 2000 Centers for Disease Control and Prevention (CDC) growth charts [27]. Underweight was defined by BMI < 5^th ^percentile, normal weight by 5th ≤ BMI < 85^th ^percentile, overweight excluding obesity by 85^th ^≤ BMI < 95^th ^percentiles, and obesity by BMI ≥ 95^th ^percentile. To complete sensitivity analyses and provide estimates comparable with other international studies, we have additionally used measures of childhood overweight and obesity based on the Cole charts for thinness (BMI centile charts reaching 17 at 18 years of age) [28] and International Obesity Task Force (IOTF) charts (BMI centile charts reaching 25 kg/m^2 ^and 30 kg/m^2 ^at 18, respectively) [29].

#### Motor skill assessments

The ECLS-B assessments of fine and gross motor skills were based on previously validated tests such as the Early Screening Inventory-Preschool or Kindergarten, the Bruininks-Oretsky Test of Motor Proficiency, and the Movement Assessment Battery for Children along with tests adapted for the sister survey ECLS-Kindergarten Cohort [26]. Before taking assessments, tests were shown to the child by the interviewer. For 4 year-old children, fine motor skill assessment evaluated the child's ability to build a tower from 10 blocks and a gate from 5 blocks. They were scored as "both passed", "one of them passed" or "none of them passed". Another fine motor measure assessed the child's ability to copy 7 shapes (e.g., lines, circle, triangle). 5-6 year-old children were asked to build a gate (assessed on a pass/fail basis) and to complete a copying exercise (4 shapes, different from the shapes assessed earlier). Each shape was scored as "pass" or "fail"; the total number of shapes successfully copied determined the copy form score (from 0 to 7 at age 4 and from 0 to 4 at age 5-6).

Gross motor skills were assessed based on the child's ability to skip at least 8 consecutive steps; walk backwards along a line for at least 6 steps; catch a bean bag tossed out of 5 trials; jump from a standing start; balance on each foot for 10 seconds and hop on each foot 5 times. All activities were demonstrated to the child by the interviewer. Except for the jump distance (measured in inches) and the number of successfully copied forms, other gross motor variables were coded on a pass/fail basis.

#### Covariates

We used information on birth, child health and behaviors, mother characteristics and family environment as covariates in multivariate regression models. Except for birth characteristics, these data were collected at each assessment in parental interviews. Some demographic characteristics such as age, race/ethnicity came from the 9-month data collection (2001-2002). Birth characteristics (weight, gestational age) came from birth certificates and pregnancy information such as mother's pre-pregnancy self-reported weight and height (to calculate pre-pregnancy BMI) and smoking during pregnancy were collected during the 9-month parental interviews. Parental self-assessment of the child's health status was collected during each interview; from 5 categories, answers were merged into 3 categories as "excellent/very good", "good", and "fair/poor". From 22 initial items, parental education was grouped into 4 categories: "no high school", "high diploma", "some college", and "college graduation". The household socioeconomic status (SES) was based on father/male and mother/female guardian's education, occupation and household income and grouped into three categories based on SES quintiles: "low SES" (1st quintile), "intermediate SES" (2nd-4th quintiles) and "high SES" (5th quintile). Finally, we created variables to describe parental involvement in child developmental activities based on the number of times parents reported going outside with children ("about once a day and more", "a few times a week", "a few times a month and less") and the frequency of reading books, singing songs and telling stories with children ("3 activities daily", "2 of the 3 activities daily", "1 of the 3 activities daily", and "no activities daily").

#### Statistical analysis

The NCES calculated survey weights to adjust for non-response and under-coverage for each round of data collection [25]. Weights and survey options ("svy") to take into account the complex sampling scheme were applied in Stata^® ^V.10.0. The sub-sample of children included in the analysis for which motor skill tests, BMI and covariates were available was compared to children with missing data for relevant differences. All analyses were stratified by gender given previously reported gender differences in the associations between body mass status and motor skills [12,13,19]. Descriptive analyses provided percentages and means and linearized standard errors of the means (SE). The association between BMI z-scores and motor skills was estimated using covariates-adjusted linear regressions for the jump distance and copy form tests, multinomial logistic models for block building tests at 4 years of age, and logistic models for the remaining dichotomous motor test variables. We also estimated associations between a categorical BMI variable (based on either the CDC references or IOTF references) and motor skills. Finally, fully-adjusted probabilities of passing motor skill tests by obese children compared to normal-weight children were calculated (ratios of probabilities are reported here). Significant two-tailed tests were set at 5%. Analyses were carried out in 2010.

## Results

### General characteristics

**Figure 1:**
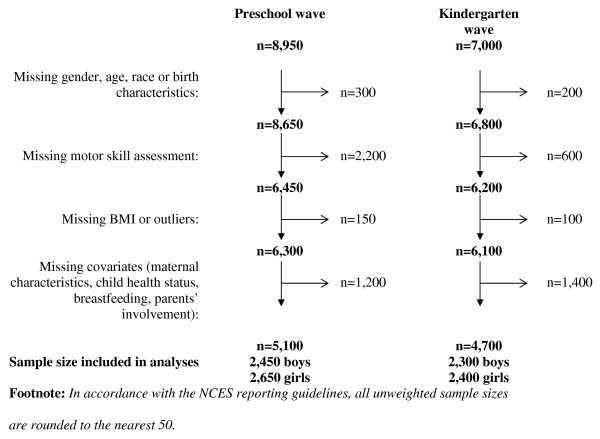
**Inclusion flow chart in analyses**. In accordance with the NCES reporting guidelines, all unweighted sample sizes are rounded to the nearest 50.

We used data for 5 100 children at the preschool assessment (out of 8 950; 57%) and 4 700 children at the kindergarten wave (out of 7 000; 67%) (Figure 1). Most of the missing data were due to lack of motor skill assessment and covariates in 4 year-old children and missing covariates in 5-6 year-old children. Child, mother and family characteristics at 4 years of age are presented in Table 1. The sample characteristics at the kindergarten assessment were almost identical to those at the preschool age (data not shown).

**Table 1:** Child, mother and family characteristics-Preschool assessment (ECLS-B cohort, 2005-2006)

	Boys n = 2 450^a^	Girls n = 2 700
**Child characteristics**		

Age (months) (mean, SE)	52.7 (0.13)	52.6 (0.10)

*Race/ethnicity (%)*		

White non-Hispanic	52.9	55.0

African American	15.8	14.1

Asian	2.4	2.6

Hispanic	24.3	23.8

Other	4.5	4.4

Prematurity (gestational age < 37 wks) (%)	11.2	11.0

*Birthweight (%)*		

< 2.5 kg	6.1	7.8

≥ 4.0 kg	10.7	5.9

*Birth rank (%)*		

Singleton	19.0	16.9

Eldest	21.5	21.7

2^d ^born	34.3	34.4

3^d ^born and more	25.1	26.9

Any breastfeeding (%)	67.5	69.6

Excellent/very good health status (%)	87.7	90.0

Television watching (hours/d) (mean, SE)	2.5 (0.07)	2.4 (0.06)

Center-based child daycare (%)	63.3	59.3

**Mother characteristics**		

Age (years) (mean, SE)	31.9 (0.22)	32.0 (0.18)

*Education (%)*		

No high school	14.6	13.2

High school diploma	27.5	26.4

Some college	30.7	32.1

College graduation	27.2	28.3

Married (%)	70.8	71.4

Overweight or obese before pregnancy (%)	39.1	39.5

Smoking during pregnancy (%)	16.7	17.0

**Family characteristics**		

Living in an urban area (%)	84.8	84.4

Low socioeconomic status (%)^b^	19.6	16.8

English spoken at home (%)	80.0	82.9

Number of children < 18 y (mean, SE)	2.4 (0.03)	2.4 (0.03)

Number of dinners as a family per wk (mean, SE)	5.5 (0.05)	5.5 (0.06)

*Parental involvement (%)^b^*		

No books/stories/songs each day	34.6	28.4

Outside walk or play few times a month or less	14.3	15.2

Estimations of percentages, means and standard errors of the mean (SE) are weighted and take into account the complex sampling design. *ECLS-B*, Early Childhood Longitudinal Survey-Birth Cohort

^a^Unweighted sample size rounded to the nearest 50. ^b^See definitions in the Methods section.

Four year-old children in the analyses were older than participants excluded due to missing data (52.7 (SE = 0.09) vs. 52.3 months (0.10), *p *< 0.01), and lived in more favorable family conditions (e.g., high SES 22.1% vs. 16.3%, *p *< 0.01). Obesity prevalence (CDC references) was statistically comparable in the analytic sample and among the excluded 4 year-old children (15.9% vs. 18.5%, *p *< 0.10), while passing gross and fine motor skill tests was more successful in the retained sample of 4 year-old children (*p *< 0.03). Five-to-six year-old children in the analysis were as old as the excluded participants (64.8 (0.08) vs. 64.7 months (0.12), *p *= 0.83) and equally affected by obesity (15.6% vs. 18.1%, *p *= 0.30), but had different living conditions (high SES 21.8% vs. 15.2%, *p *< 0.01) and lower motor skills (p < 0.01, except for the skip test: *p *= 0.52).

According to the CDC growth charts, one third of the children were classified as overweight or obese and around 15% of children were obese (Table 2). Average rates of successfully passing motor skill tests varied from 20% to 80%, depending on the test type and children's age (Table 2). Average gross motor skills were higher in 5-6 year-old than in 4 year-old children. Girls were on average more successful than boys in passing the balance, skip, copying, hop on the right foot and walk backwards tests (p < 0.01), but their jump distance was usually lower than in boys (p < 0.01).

**Table 2:** Distribution of body mass index (BMI) z-score, BMI categories and motor skills

	Boys		Girls	
	**4 y** **n = 2 450^a^**	**5-6 y** **n = 2 300**	**4 y** **n = 2 700**	**5-6 y** **n = 2 400**

**Body mass index (BMI)**				

Z-score (mean, SE)	0.62 (0.03)	0.63 (0.03)	0.63 (0.03)	0.65 (0.03)

*CDC categories (%)*				

Underweight	2.2	1.9	1.8	1.9

Normal	63.8	65.1	63.6	64.1

Overweight	17.3	17.2	19.5	18.7

Obesity	16.8	16.1	15.1	15.1

*IOTF and Cole categories^b ^(%)*				

Underweight	0.9	0.5	0.8	0.5

Normal	77.1	75.9	73.5	70.1

Overweight	15.7	16.6	17.2	19.0

Obesity	6.3	7.0	8.5	10.4

**Motor skill^c^**				

**Gross motor skill**				

*Balance at least 10 sec. (%)*				

Right foot	45.8	75.0	53.6	83.3

Left foot	44.6	74.4	52.5	81.9

*Hop 5 times (%)*				

Right foot	66.2	88.1	70.4	92.2

Left foot	61.3	85.5	63.9	89.8

Jump distance (inches) (mean, SE)	27.9 (0.24)	32.4 (0.28)	24.7 (0.28)	29.9 (0.31)

Skip at least 8 steps (%)	20.5	37.7	32.4	59.2

Walk backwards 6 steps on line (%)	33.9	38.3	40.9	48.6

Bean bag catch (at least 5 caught) (%)	42.1	51.8	38.3	50.8

**Fine motor skill^c^**				

Blocks (%)				

Passed one (either tower or gate)	44.1	-	44.9	-

Passed both	42.9	-	42.9	-

Gate passed	-	79.2	-	81.8

Copy form (mean, SE)	3.2 (0.04)	2.5 (0.04)	3.8 (0.04)	2.3 (0.04)

Estimations of percentages, means and standard errors of the mean (SE) are weighted and take into account the complex sampling design.

*BMI*; Body mass index, *CDC*; Centers for Disease Control and prevention, *IOTF*; International Obesity Task Force

^a^Unweighted sample size rounded to the nearest 50. ^b^See definitions in the Methods section. ^c^Percentages of passing the test are presented (except for jump distance).

### Association between BMI z-score, obesity and motor skills

The only motor skill measure that consistently varied with weight status in boys and girls was hopping. Specifically, BMI z-score was inversely associated with passing the hop test in boys (left foot at 4 years of age and right foot at 5-6 years) and among 5-6 year-old girls (Table 3). Other motor skill assessments had no detectable association with children's body weight or did so only in certain age-gender groups. For example, girls with higher BMI z-scores had on average a lower jump distance at both 4 and 5-6 years of age, but boys showed no difference. There was also a positive result for heavier body weight: 4 year-old girls with higher BMI z-scores had a higher frequency of passing the bean bag catching test. Using categorical variables of BMI, obese boys and girls were about 17-20% less likely to pass the hop test compared to normal-weight children at 4 years of age and 7-11% at 5-6 years (Table 3). It was the case for both feet in boys and for the left foot in girls. In addition, obese girls had a shorter jump distance than normal weight girls at both survey waves.

**Table 3:** Association of body mass index (BMI) z-score and obesity with motor skills

	Boys		Girls	
	**BMI z-score**	**Obesity**	**BMI z-score**	**Obesity**

**4 years of age**				

**Gross motor skill**				

*Balance at least 10 sec*.				

Right foot	-0.07	0.84	-0.05	0.92

Left foot	-0.04	0.94	-0.05	0.77**

*Hop 5 times*				

Right foot	-0.06	0.83**	-0.03	0.91

Left foot	-0.07*	0.80**	-0.08	0.83**

Jump distance (inches)	-0.40	-1.04	-0.46*	-1.69**

Skip at least 8 steps	-0.01	0.84	0.04	1.03

Walk backwards 6 steps on line	-0.01	0.91	-0.05	0.93

Bean bag catch (at least 5 caught)	0.03	1.14	0.08*	1.32**

**Fine motor skill**				

Blocks (gate & tower)				

Passed one	0.002	1.00	0.04	1.03

Full passed	-0.003	0.99	0.04	0.94

Copy form	-0.02	-0.10	-0.02	0.00

**5-6 years of age**				

**Gross motor skill**				

*Balance at least 10 sec*.				

Right foot	-0.02	0.94	-0.01	1.02

Left foot	0.05	0.99	-0.09*	0.98

*Hop 5 times*				

Right foot	-0.12*	0.92*	-0.08	0.96

Left foot	-0.05	0.89**	-0.15**	0.93*

Jump distance (inches)	-0.15	-1.07	-0.47*	-1.58**

Skip at least 8 steps	0.07	0.99	-0.04	0.97

Walk backwards 6 steps on line	0.04	0.92	-0.08*	0.91

Bean bag catch (at least 5 caught)	0.07	1.06	-0.01	0.98

**Fine motor skill**				

Blocks (gate)	0.06	0.97	0.04	1.06

Copy form	0.04	0.05	-0.01	-0.03

Values are regression coefficients for BMI z-score and ratio of probabilities for passing a test in obese children out of normal-weight children. All analyses are adjusted for covariates and take into account weights and sampling design. *P < 0.05; **P < 0.01

No difference in motor skills was observed in overweight (not obese) children compared to normal weight children, except for a higher probability of passing the bean bag test (probability ratio = 1.20, *p *< 0.05) and a lower copying form score (coef. = -0.18; *p *< 0.05) in overweight 4 year-old boys compared to normal-weight counterparts. Sensitivity analyses using the obesity IOTF references showed the same patterns with motor abilities as with the CDC-based thresholds, also including a lower jump distance in obese 4 year-old boys (linear regression coefficient: -2.1, *p *< 0.05) and a lower probability of passing balance tests in obese compared to normal-weight 4 year-old children (right foot in boys: probability ratio = 0.72, *p *< 0.05; left foot in girls: probability ratio = 0.82; *p *< 0.05).

## Discussion

Based on the U.S. nationally representative data, most motor skills are not impaired in obese or overweight children of 4 and 5-6 years of age. Only gross motor skills that seem to be directly influenced by a child's heavy body weight, such as hopping in boys and girls and a jump distance in girls, were inversely associated with obesity and higher BMI z-scores. Motor skills involving coordination, balance and control were not lower in children with higher BMI. Fine motor skills were not related to obesity and BMI of children ages 4 to 6.

### Fine motor skills and BMI

Our finding of no significant association between obesity (or BMI z-scores) and fine motor skills at 4 to 6 years of age is consistent with previous research looking at fine motor skills [15]. It is possible that general motor skill impairments showed in studies assessing fine and gross motor skills without distinction (i.e. combined in one measure) [12,13,22] reflect the effect of gross motor skill impairment in high-BMI young children. One study that examined the link between specifically fine motor skills and obesity found a significant association in 9-13 year-old children but not in 5-9 year-olds [20]. Acquisition of fine motor skills occurs throughout childhood, so differences in skills of certain risk groups may become apparent later in childhood when skills become more complex and diversified. Prevention of fine motor impairment in early childhood is important so that all children have the same chance for successful development. The mechanisms by which fine motor skills decrease with increasing BMI in children when they become older need better understanding.

### Gross motor skills and BMI

The association between gross motor skills and body mass status of 4-year-old and 5-6-year-old children varied by type of skills, with some differences observed across gender and age groups. Our findings of diminished hopping and jumping skills with higher BMI z-scores and obesity are consistent with results shown in previous studies [16,18,19,30-32]. One study examined the link between body weight and running ability and found that obese children were not able to run as quickly as their non-obese peers [16]. Such locomotor competences are likely to be directly related to the excess weight and impaired musculoskeletal functions of obese children [21]. The finding that the jumping ability was associated with obesity among girls only (as also found in another study) [33] may be partly interpreted in relation to BMI specificity (between 85% to 95% according to the studies) [34,35] that could lead to misclassification of some muscular physically active boys as overweight or obese. This explanation is indeed plausible since the jump distance correlated with obesity in boys using the IOTF references. The IOTF thresholds are higher than the CDC 95^th ^percentiles at early ages, and may have lower rates of misclassification of muscular boys. This also highlights the likely role of muscular development in reducing the gap in motor skills due to body weight status and helps us understand some apparent discrepancies across our findings. At last, different abilities between boys and girls may also reflect differences in physical games that they play, even though the impact of such choices has not been documented.

Jumping and hopping are skills used in activities with relatively high energy expenditure. Limitations of these skills may lead to lower engagement of obese children in sports and physical activity that involve jumping or hopping [36], which may further contribute to sustainability of excessive body weight and even further fat accumulation [37]. Schools should identify physical activities adapted to children's respiratory fitness and body mass status to prevent injury [38]. Motor skill abilities such as balancing, walking backwards and catching were generally of the same level in obese and normal-weight children of 4-6 years of age. Participation of obese children in sports that involve such skills should be encouraged in order to prevent obesity-associated differences in gross motor skills in later childhood and adolescence [39,40], as well as for social interactions and self-esteem development.

### Strengths and limitations

This study contributes to the literature by providing reliable estimates of the association between body weight and motor skills in 4 and 5-6 year-old children. Drawing from a nationally-representative sample of U.S. children, objective measures of child motor skills and body weight were used and associations were studied accounting for individual and family environment characteristics. Indeed, the aim was to control for a maximum of potential confounding factors. However, this study has some limitations. First, using a sub-sample with complete data has likely led to selection bias despite calibration on the national census using the survey weights. Since children in analysis were of almost the same obesity status yet exhibited higher motor skills than the excluded participants, we may underestimate the strength of the observed associations. Our estimates can also be attenuated by the fact that children in our analytic sample lived in more favourable conditions than children excluded from the analyses. Still, most of the children's characteristics in the analytic sample were similar to national estimates for the same birth cohort [41,42]. Furthermore, assessing multiple measures of various motor skills separately (no overall motor score was available in this survey) might have contributed to some ambiguity about results that need further investigation. In addition, the choice of motor skill tests can be debated since no definitive consensus on the best measurement exists in this field. Moreover, to facilitate interpretation of results, we used the pass/fail variable to describe motor skills, especially for gross motor skills. This could have led to lower sensitivity of our tests to detect differences between groups. However, using test scores as a continuous variable did not change results (data not shown). The cross-sectional design of the analysis limits causal interpretations; a longitudinal study of the impact of early childhood overweight and obesity on future motor skills would be a valuable contribution to existing knowledge on this topic.

## Conclusions

Child motor skills are adversely associated with obesity and BMI z-scores only for skills most directly related to body weight, such as jumping and hopping. Fine motor skills and skills involving coordination do not seem to correlate with obesity in 4 to 6 year olds. This study used a large national sample of young children with comprehensive objective evaluation of children's motor skills and body weight. Future analyses of large longitudinal samples should enable better understanding of such relationships and interactions between the determinants of childhood overweight and obesity and motor skills, including the issue of reverse causality. Finally, physical activity interventions designed to build upon obese children's physical strengths and encourage successful activity experiences are needed.

## Abbreviations

BMI: Body Mass Index; CDC: Centers for Disease Control; ECLS-B: Early Childhood Longitudinal Survey-Birth; IOTF: International Obesity task Force; NCES: National Center for Education Statistics; NCHS: National Center for Health Statistics; OHRP: Office for Human Research Protections; SE: Standard Error of the means; SES: Socioeconomic status.

## Competing interests

The authors declare that they have no competing interests.

## Authors' contributions

KC conceived the analyses design, performed statistical analyses, interpreted the results and wrote the manuscript. TA substantially contributed to the analyses design conception, results interpretation and writing of the manuscript. Both authors read and approved the final manuscript.

## Authors' information

KC was a visiting researcher at the Rudd Center for Food Policy and Obesity at the time of the research.

## Pre-publication history

The pre-publication history for this paper can be accessed here:

http://www.biomedcentral.com/1471-2431/12/28/prepub

## References

[B1] Ogden CL Carroll MD Curtin LR Lamb MM Flegal KM Prevalence of high body mass index in US children and adolescents, 2007-2008 JAMA 2010 303 242 249 10.1001/jama.2009.2012 20071470

[B2] Lissner L Sohlstrom A Sundblom E Sjoberg A Trends in overweight and obesity in Swedish schoolchildren 1999-2005: has the epidemic reached a plateau? Obes Rev 2010 11 553 559 20025696 10.1111/j.1467-789X.2009.00696.x

[B3] Olds TS Tomkinson GR Ferrar KE Maher CA Trends in the prevalence of childhood overweight and obesity in Australia between 1985 and 2008 Int J Obes (Lond) 2010 34 57 66 10.1038/ijo.2009.211 19823187

[B4] Salanave B Peneau S Rolland-Cachera MF Hercberg S Castetbon K Stabilization of overweight prevalence in French children between 2000 and 2007 Int J Pediatr Obes 2009 4 66 72 10.1080/17477160902811207 19306152

[B5] Troiano RP Flegal KM Overweight children and adolescents: description, epidemiology, and demographics Pediatrics 1998 101 497 504 12224656

[B6] Lopes VP Stodden DF Bianchi MM Maia JA Rodrigues LP Correlation between BMI and motor coordination in children J Sci Med Sport 2012 15 38 43 10.1016/j.jsams.2011.07.005 21831708

[B7] Gortmaker SL Must A Perrin JM Sobol AM Dietz WH Social and economic consequences of overweight in adolescence and young adulthood N Engl J Med 1993 329 1008 1012 10.1056/NEJM199309303291406 8366901

[B8] Li W Rukavina P A review on coping mechanisms against obesity bias in physical activity/education settings Obes Rev 2009 10 87 95 10.1111/j.1467-789X.2008.00528.x 18828779

[B9] Gale CR Batty GD Cooper C Deary IJ Psychomotor coordination and intelligence in childhood and health in adulthood-testing the system integrity hypothesis Psychosom Med 2009 71 675 681 10.1097/PSY.0b013e3181a63b2e 19483120

[B10] Osika W Montgomery SM Physical control and coordination in childhood and adult obesity: Longitudinal Birth Cohort Study BMJ 2008 337 a699 10.1136/bmj.a699 18698093 PMC2769521

[B11] Lubans DR Morgan PJ Cliff DP Barnett LM Okely AD Fundamental movement skills in children and adolescents: review of associated health benefits Sports Med 2010 40 1019 1035 10.2165/11536850-000000000-00000 21058749

[B12] Cairney J Hay JA Faught BE Hawes R Developmental coordination disorder and overweight and obesity in children aged 9-14 y Int J Obes (Lond) 2005 29 369 372 10.1038/sj.ijo.0802893 15768042

[B13] Cawley J Spiess CK Obesity and skill attainment in early childhood Econ Hum Biol 2008 6 388 397 10.1016/j.ehb.2008.06.003 18678531

[B14] Cliff DP Okely AD Morgan PJ Jones RA Steele JR Baur LA Proficiency Deficiency: Mastery of Fundamental Movement Skills and Skill Components in Overweight and Obese Children Obesity (Silver Spring) 2011 (doi:10.1038/oby.2011.241) 10.1038/oby.2011.241 21799480

[B15] D'hondt E Deforche B De BI Lenoir M Relationship between motor skill and body mass index in 5- to 10-year-old children Adapt Phys Activ Q 2009 26 21 37 19246771 10.1123/apaq.26.1.21

[B16] Graf C Koch B Kretschmann-Kandel E Falkowski G Christ H Coburger S Correlation between BMI, leisure habits and motor abilities in childhood (CHILT-project) Int J Obes Relat Metab Disord 2004 28 22 26 10.1038/sj.ijo.0802428 14652619

[B17] Morano M Colella D Caroli M Gross motor skill performance in a sample of overweight and non-overweight preschool children Int J Pediatr Obes 2011 6 Suppl 2 42 46 21923296 10.3109/17477166.2011.613665

[B18] Poulsen AA Desha L Ziviani J Griffiths L Heaslop A Khan A Fundamental movement skills and self-concept of children who are overweight Int J Pediatr Obes 2011 6 e464 e471 10.3109/17477166.2011.575143 21627397

[B19] Mond JM Stich H Hay PJ Kraemer A Baune BT Associations between obesity and developmental functioning in pre-school children: a population-based study Int J Obes (Lond) 2007 31 1068 1073 10.1038/sj.ijo.0803644 17471298

[B20] D'hondt E Deforche B De BI Lenoir M Childhood obesity affects fine motor skill performance under different postural constraints Neurosci Lett 2008 440 72 75 10.1016/j.neulet.2008.05.056 18541379

[B21] Wearing SC Hennig EM Byrne NM Steele JR Hills AP The impact of childhood obesity on musculoskeletal form Obes Rev 2006 7 209 218 10.1111/j.1467-789X.2006.00216.x 16629876

[B22] Slining M Adair LS Goldman BD Borja JB Bentley M Infant overweight is associated with delayed motor development J Pediatr 2010 157 20 25 10.1016/j.jpeds.2009.12.054 20227724 PMC3395373

[B23] D'hondt E Gentier I Deforche B Tanghe A De BI Lenoir M Weight loss and improved gross motor coordination in children as a result of multidisciplinary residential obesity treatment Obesity (Silver Spring) 2011 19 1999 2005 10.1038/oby.2011.150 21720438

[B24] Bethel J Green J Kalton G Nord C Early Childhood Longitudinal Study, Birth Cohort (ECLS-B), sampling. Vol 2 of the ECLS-B Methodology Report for the 9-Month Data Collection, 2001.02 (NCES 2005.147) 2005 Washington, DC, US Department of Education, National Center for Education Statistics

[B25] Wheeless S Ault K Copello E Black S Johnson R Early Childhood Longitudinal Study, Birth Cohort (ECLS-B), Methodology Report from the Kindergarten 2006 Data Collection (2006-07), Volume II: Sampling (NCES 2010-07) 2010 Washington, DC, National Center for Education Statistics, Institute of Education Sciences, U.S. Department of Education 1 402

[B26] Najarian M Snow K Lennon J Kinsey S Early Childhood Longitudinal study, Birth Cohort (ECLS-B), Preschool-Kindergarten 2007 Psychometric Report (NCES 2010-009) 2010 National Center for Education Statistics, Institute of Education Science, U.S., Department of Education. Washington, DC http://nces.ed.gov/pubs2010/2010009.pdf 6-10-2010

[B27] Kuczmarski RJ Ogden CL Guo SS Grummer-Strawn LM Flegal KM Mei Z 2000 CDC Growth Charts for the United States: methods and development Vital Health Stat 2002 11 1 190 12043359

[B28] Cole TJ Flegal KM Nicholls D Jackson AA Body mass index cut offs to define thinness in children and adolescents: international survey BMJ 2007 335 194 10.1136/bmj.39238.399444.55 17591624 PMC1934447

[B29] Cole TJ Bellizzi MC Flegal KM Dietz WH Establishing a standard definition for child overweight and obesity worldwide: international survey BMJ 2000 320 1240 1243 10.1136/bmj.320.7244.1240 10797032 PMC27365

[B30] Cliff DP Okely AD Magarey AM Movement skill mastery in a clinical sample of overweight and obese children Int J Pediatr Obes 2011 6 473 475 10.3109/17477166.2011.575154 21609208

[B31] Jones RA Okely AD Gregory P Cliff DP Relationships between weight status and child, parent and community characteristics in preschool children Int J Pediatr Obes 2009 4 54 60 10.1080/17477160802199984 18608633

[B32] Morano M Colella D Robazza C Bortoli L Capranica L Physical self-perception and motor performance in normal-weight, overweight and obese children Scand J Med Sci Sports 2010 10.1111/j.1600-0838.2009.01068.x 20136752

[B33] Jones RA Okely AD Caputi P Cliff DP Relationships between child, parent and community characteristics and weight status among young children Int J Pediatr Obes 2010 5 256 264 10.3109/17477160903271971 19900149

[B34] Laurson KR Eisenmann JC Welk GJ Body Mass Index standards based on agreement with health-related body fat Am J Prev Med 2011 41 S100 S105 10.1016/j.amepre.2011.07.004 21961608

[B35] Mei Z Grummer-Strawn LM Pietrobelli A Goulding A Goran MI Dietz WH Validity of body mass index compared with other body-composition screening indexes for the assessment of body fatness in children and adolescents Am J Clin Nutr 2002 75 978 985 12036802 10.1093/ajcn/75.6.978

[B36] Deforche B De BI D'hondt E Cardon G Objectively measured physical activity, physical activity related personality and body mass index in 6- to 10-yr-old children: a cross-sectional study Int J Behav Nutr Phys Act 2009 6 25 10.1186/1479-5868-6-25 19442293 PMC2690577

[B37] Parsons TJ Power C Logan S Summerbell CD Childhood predictors of adult obesity: a systematic review Int J Obes Relat Metab Disord 1999 23 Suppl 8 S1 S107 10641588

[B38] Floriani V Kennedy C Promotion of physical activity in primary care for obesity treatment/prevention in children Curr Opin Pediatr 2007 19 99 103 10.1097/MOP.0b013e328013c88c 17224670

[B39] D'hondt E Deforche B Vaeyens R Vandorpe B Vandendriessche J Pion J Gross motor coordination in relation to weight status and age in 5- to 12-year-old boys and girls: a cross-sectional study Int J Pediatr Obes 2011 6 e556 e564 10.3109/17477166.2010.500388 20973659

[B40] Okely AD Booth ML Chey T Relationships between body composition and fundamental movement skills among children and adolescents Res Q Exerc Sport 2004 75 238 247 15487288 10.1080/02701367.2004.10609157

[B41] Martin JA Hamilton BE Ventura SJ Menacker F Park MM Sutton PD Births: final data for 2001 Natl Vital Stat Rep 2002 51 1 102 12596439

[B42] Ogden CL Carroll MD Flegal KM High body mass index for age among US children and adolescents, 2003-2006 JAMA 2008 299 2401 2405 10.1001/jama.299.20.2401 18505949

